# Walking the Talk for Dementia: A Catalyst For Change in the Global Dementia Landscape

**DOI:** 10.1093/geroni/igaf122.2235

**Published:** 2025-12-31

**Authors:** Walter Dawson, Sherril Gelmon, Fernando Aguzzoli Peres, Jonathan Adrian Zegarra Valdivia, Kate Irving, Lingani Mbakile-Mahlanza

**Affiliations:** Oregon Health & Science University, Portland, Oregon, United States; Portland State University, Portland, Oregon, United States; Blobal Brain Health Institute, Dublin, Dublin, Ireland; Achucarro Basque Center for Neuroscience, Bilbao, Pais Vasco, Spain; Dublin City University, Dublin, Dublin, Ireland; University of Botswana, Goborone, South-East, Botswana

## Abstract

Walking the Talk for Dementia (WTD) is an innovative global initiative that challenges the stigma surrounding dementia, fosters intergenerational and multidisciplinary dialogue, and inspires a renewed sense of purpose among participants. The annual event combines a four-day, 40 km pilgrimage to Santiago de Compostela, Spain, with a two-day symposium, creating an immersive space to reflect on the physical, emotional, spiritual, and societal dimensions of dementia. WTD prioritizes multicultural and multistakeholder engagement, elevating the voices and improving the quality of life of people living with dementia, care partners, and advocates while promoting inclusive and sustainable solutions for global brain health. WTD integrates professional and lived experiences, fosters collaboration to address stigma, increase awareness, and build an empathetic community. Data collection occurred before, during, and after the 2024 WTD through surveys and personal reflections from participants (n = 79) representing 27 countries. Post-event surveys demonstrated high levels of satisfaction. Participants rated the effectiveness of the unique WTD format highly, and reported the experience as superior to the traditional academic conference model regarding networking and engaging with people with lived experience. Thematic analysis of qualitative responses highlighted the transformative nature of WTD. Walking the Talk for Dementia exemplifies how dementia changemakers across disciplines and regions can be inspired. By integrating physical activity, interpersonal dialogue, and potentially emotional experiences, WTD empowers participants to transform stigma into understanding, and challenges them to create opportunities for global advocacy. Participants’ voices underscore its profound impact, identifying WTD as a powerful catalyst for change in the global dementia landscape.

